# A Facile Synthesis of Polypyrrole/Carbon Nanotube Composites with Ultrathin, Uniform and Thickness-Tunable Polypyrrole Shells

**DOI:** 10.1186/1556-276X-6-431

**Published:** 2011-06-17

**Authors:** Bin Zhang, Yiting Xu, Yifang Zheng, Lizong Dai, Mingqiu Zhang, Jin Yang, Yujie Chen, Xudong Chen, Juying Zhou

**Affiliations:** 1 Key Lab Polymer Composite & Funct Mat, Key Lab Designed Synth & Applicat Polymer Mat, School of Chemistry and Chemical Engineering, Sun Yat-Sen University, Guangzhou, 510275, China; 2 College of Chemistry and Chemical Engineering, State Key Laboratory for Physical Chemistry of Solid Surfaces, Xiamen University, Xiamen, 361005, China; 3 Institute of Photonics, SUPA, University of Strathclyde, Glasgow G4 0NW, UK

## Abstract

An improved approach to assemble ultrathin and thickness-tunable polypyrrole (PPy) films onto multiwall carbon nanotubes (MWCNTs) has been investigated. A facile procedure is demonstrated for controlling the morphology and thickness of PPy film by adding ethanol in the reaction system and a possible mechanism of the coating formation process is proposed. The coated PPy films can be easily tuned by adding ethanol and adjusting a mass ratio of pyrrole to MWCNTs. Moreover, the thickness of PPy significantly influences the electronic conductivity and capacitive behavior of the PPy/MWCNT composites. The method may provide a facile strategy for tailoring the polymer coating on carbon nanotubes (CNTs) for carbon-based device applications.

## Introduction

Over the last two decades, carbon nanotubes (CNTs) have been widely used as fillers in desirable combinations with functional polymers because of their high electrical conductivity, chemical stability, low mass density, and large surface area [1-3]. Composite materials of CNTs and polymers have attracted great interest because they may possess novel combinations with superior characteristics than either of the individual components [4-9]. Among them, it has been already confirmed that the composites consisted of electronically conducting polymers (ECPs) and CNTs possess the superior electrical properties than either of the individual components [8], which are potential materials for the development of organic electronic devices, such as organic photovoltaic cells, [10] biologic sensors [11] and flexible light-emitting diodes[12]. To the best of our knowledge, the interfacial structure between nanotube and polymer including the morphology and thickness of polymer is critical to tailor their structures and properties in many potential applications[13].

So far, a variety of methods such as chemical oxidation process, electrochemical or chemical polymerization through surfactants and template synthesis [14-19] have been investigated for producing composites from the combination of CNTs with conducting polymers. Unfortunately, CNTs have often been coated with thick and nonuniform layers, which range from 50 to 80 nm [14-18], and encapsulated aggregation of CNTs within the bulk polymer matrix due to the poor solubility of CNTs and partial exfoliation of nanotube bundles[20]. Moreover, successful results of the PPy/CNT composites with tunable thickness of the polymer shell have rarely been obtained [21,22]. The major problem exists in the processibility of CNTs in solution and the controll of interfacial bonding in polymer/CNTs composites. Due to the hydrophobic nature and strong van der Waals interactions between CNTs, as-produced CNTs pack into crystalline ropes and tangle networks which are found to act as an obstacle to most applications, especially diminishing the special mechanical and electrical properties of the individual tubes [23]. Furthermore, inherently weak nanotube-polymer interactions result in the poor interfacial adherent [24], which will lead to the agglomeration of conjugated polymers. The polymer chains incline to form deposits of irregular nanoparticles or sediments with a diameter of about 50 nm[19,20,23]. Consequently, one way to overcome these limitations is to control the polymerization rate of the pyrrole monomers and improve the processibility of CNTs in solution.

Herein, we report a facile approach to assemble ultrathin and uniform PPy films onto multiwall carbon nanotubes [MWCNTs] to form a one-dimensional hybrid nanostructure by an improving *in situ *chemical oxidation polymerization. The addition of ethanol in the aqueous reaction system is a key point for tuning the morphology and thickness of PPy shell by controlling the polymerization rate [24], which overcomes the significant challenge in enhancing the interfacial bonding between polymer and carbon nanotubes. The PPy/MWCNT composites possess the core (individual MWCNT)/shell (PPy film) structure and no agglomerations or irregular nanoparticles of polymer are found on the surface of the composites. Furthermore, the synthesis process does not need any surfactant assistance and the thickness of the polymer shell can be precisely controlled by adding ethanol and changing the mass ratio of PPy/MWCNT. Moreover, the influences of the thickness of coating-polymer on the electrical properties of the PPy/MWCNT composites have been explained systemically. The results can provide the basis for tuning the polymer thickness to improve the properties of carbon-based device.

## Results and Discussion

**Figure 1:**
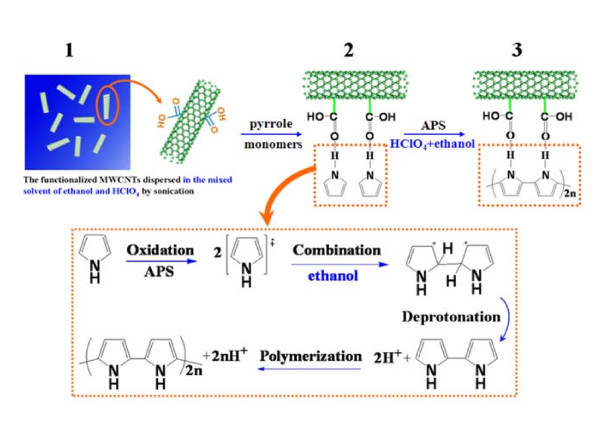
**Diagram of synthesis process for PPy/MWCNT composites**.

The preparation of the PPy/MWCNT composites based on a improved *in situ *chemical oxidation polymerization method which can be expressed in Figure 1. The surface modification of MWCNTs was performed with carboxylic acid groups yielding MWCNT-COOH. Importantly, two points should be noted in the improved reaction process: **1) **The adding sequence of monomer and initiator is an effective way to achieve polymerization in the desired locations. The carboxylic acid groups are likely to offer the interfacial interaction between the polymer and the nanotubes due to the hydrogen bonds formed between -COOH groups of chemically modified MWCNTs and NH groups of the PPy[21]. The contact junctions between MWCNTs and PPy films can be remarkablely improved by avoiding the use of insulating surfactants and other organic solutions. **2) **The CNTs easily precipitate into ropes or bundles due to the hydrophobic nature and strong van der Waals interactions between CNTs. So the homogeneous dispersion of nanotubes in solution with high surface area is particularly important. Ethanol is added in aqueous solution which is beneficial to well disperse the tubes and stabilize the MWCNTs to prevent agglomerations or precipitate. Moreover, ethanol is often used as the free radical collecting agent which exhibits a restraint effect on the polymerization reaction. The polymerization rate of pyrrole monomers is reduced by adding ethanol, this can control the self polymerization of pyrrole monomers and favor the even attachment of polymer film on the MWCNTs surface. It is clearly shown in the low resolution typical transmission electron microscopy (TEM) images (see Figure S1 in Additional file 1), compared with that prepared without adding ethanol, carbon nanotubes are better dispersed and not randomly entangled in the PPy/MWCNT composites by adding ethanol in solution. In addition, the surface of the PPy/MWCNT composites appears to be smooth and uniform, and no agglomerations or irregular nanoparticles of polymer are found. The various ratio of ethanol and acid solution as the reaction solution significantly influence the morphology of the PPy/MWCNT composites. The detail of synthesis process is described in ESM. The PPy films coating on the surface of CNTs synthesized in the mixed solution of V_ethanol_/V_acid solution _= 1:1 are smoother and more uniform compared with those obtained in the V_ethanol_/V_acid solution _= 1:5 solution, but the reaction time is prolonged markedly [24] (TEM images as shown in Figure S2 in Additional file 1). It proves our conjecture that the ethanol can effectively reduce the polymerization rate of PPy.

**Figure 2:**
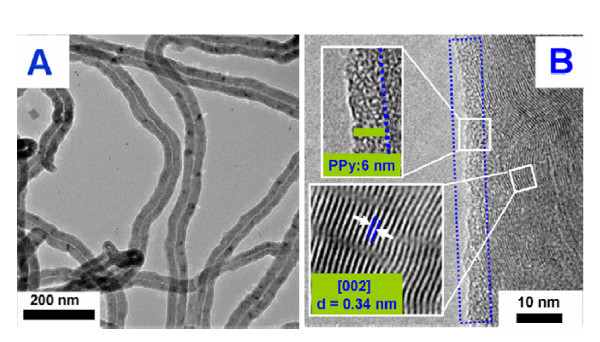
**(A) Typical TEM and (B) HRTEM images of PPy/MWCNT composites (the mass ratio of PPy/MWCNT is 2:8)**.

TEM image of PPy/MWCNT (2:8) composites is shown in Figure 2A. The image reveals a coaxial structure of the resulted PPy/MWCNT composites in which the MWCNT is encapsulated by a uniform shell of PPy. Figure 2B shows an high-resolution TEM image of a segment of MWCNT coated with the ultrathin polymer shell. The original MWCNT core with a crystalline lattice structure and an amorphous PPy coating layer can be clearly identified. The MWCNT has an interlayer spacing of 0.34 nm, which corresponds to the interplanar distance of (002) planes of graphite. Importantly, the thickness of the PPy is about 6 nm, which reveals the close interfacial contact between the PPy layer and MWCNTs[25,26].

**Figure 3:**
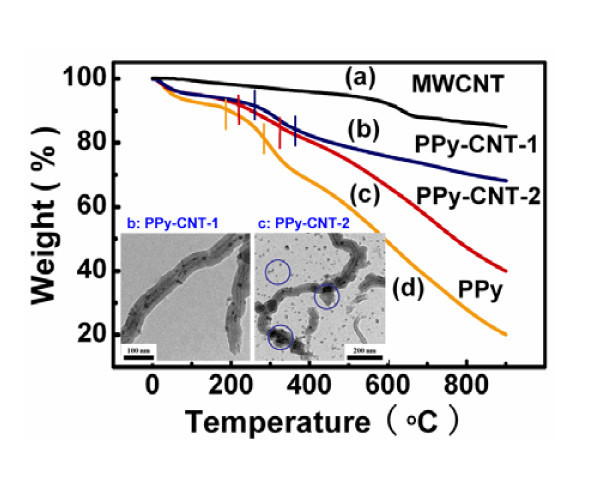
**TGA analysis of PPy, MWNT, and PPy/MWCNT composites: (a) MWCNT-COOH; (b) PPy/MWCNT-1 composite prepared by adding ethanol; (c) PPy/MWCNT-2 composite prepared without ethanol; (d) pure PPy**. The insets are the TEM images PPy/MWCNT-1 and PPy/MWCNT-2, respectively.

Changes in intrinsic polymer properties brought about by the addition of MWCNTs are indicative of nanotube-matrix interactions [22]. Improved thermal stability in polymer/CNTs composites systems relative to the polymers have been predicted by classical molecular dynamic simulations [27]. Therefore, thermogravimetric analyzer measurements of the PPy/MWCNT composites were carried out, and the results are shown in Figure. 3. MWCNTs are comparatively stable and showing no dramatic decomposition, with a 15% mass loss being observed [21]. However, for pure PPy, two steps rapid mass loss occurred at around 190°C and 320°C are depicted by two vertical lines in curve d, which is attributed to the thermal oxidative decomposition of PPy chains, and only 20% mass remained for pure PPy at 900°C [28]. For investigating the thermal oxidative decomposition of PPy/MWCNT composites with different shell thickness, two PPy/MWCNT composites were prepared at the same mass ratio of pyrrole to MWCNTs (6:4) with (PPy-CNT-1, curve b) and without (PPy-CNT-2, curve c) the addition of ethanol in the reaction solution. Two steps rapid mass loss are also observed as indicated by the vertical lines in curves b and c, respectively [29] These two composites show more delay decomposition compared to pure PPy. The improved thermal stability of PPy/MWCNT composites indicates that there should exist interfacial interaction between CNTs with polymer shell[21]. Furthermore, it is worth noting that the temperatures of two steps rapid mass loss for PPy-CNT-1 composite (curve b) are increased from 210°C and 360°C to 280°C and 410°C, respectively, compared with the PPy-CNT-2 composite (curve c). In contrast, the coated-polymer for the PPy-CNT-2 composite is 20 wt.% higher than the former. The reason for this is given by the TEM images of the two PPy/MWCNT composites (shown in the inset of Figure 3). Both of the two images reveals a coaxial structure of the resulted PPy/MWCNT composites in which the MWCNT is encapsulated by a uniform shell of PPy. However, the surface of PPy-CNT-1 composite appears to be smooth and uniform, and there are no agglomerations or irregular nanoparticles of polymer after a sonicated dispersion. Clearly, a lot of irregular PPy particles and some agglomerations are found when PPy/MWCNT composite is fabricated without ethanol. The granular PPy products absorbed on the carbon nanotubes surface during the ragid self polymerization reaction of PPy exhibit the weak adherent ability to the carbon nanotubes. Therefore, ethanol plays an important role in restraining the polymerization reaction, controlling the self polymerization of pyrrole monomers, enhancing the interfacial bonding of polymer/carbon nanotubes and controlling the morphology of polymer film on MWCNTs surface.

**Figure 4:**
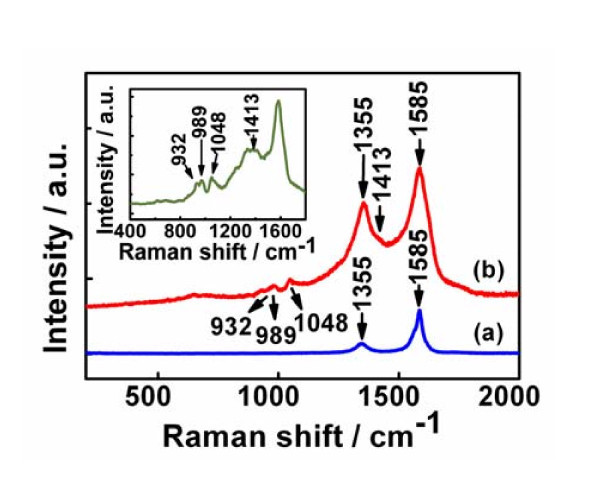
**Room-temperature Raman spectra of (a) pristine MWCNT and (b) PPy/MWCNT composite**. The inset curve is the Raman spectrum of pure PPy.

Raman spectroscopy has also been used to investigate the surface and interfacial properties of PPy/carbon nanotubes composites [30]. From the room temperature Raman spectra of (a) MWCNT and (b) PPy/MWCNT (2:8) composites (Figure 4), we can see that the typical peak of pristine MWCNT (Figure. 4a) at 1,591 cm^-1 ^(G-band) is attributed to E_2 g _mode of graphite wall. The band at 1,334 cm^-1 ^(D-band) is assigned to slightly disordered graphite [30]. Clearly, after the shell coating forms on MWCNTs surface, four additional Raman peaks (appeard at around 932, 989, 1,048, and 1,413 cm^−1^, respectively) are found. From the Raman spectra of pure PPy (inset curve in Figure 4), the bands at approximately 932 and 989 cm^−1 ^are assigned to the ring deformation associated with the di-cation (di-polaron) and radical cation (polaron), respectively [31]. The band at approximately 1,413 cm^−1 ^can be attributed to the C-N stretching mode and the peak at around 1,048 cm^−1 ^to the C-H in plane deformation [32]. The G-band and D-band of MWCNT clearly change with PPy coating, demonstrating the interfacial interactions between the MWCNT and PPy [31]. Interestingly, polaron mode shifted from in 1,048 cm^-1 ^of pure PPy to 1,051 cm^-1 ^of PPy/MWNT array and the peak intensity increases compared with that of the peak at 989 cm^−1^, and the high frequency C-H in-plane deformation mode at 1051 cm^-1 ^is correlated with the high electric conductivity of PPy [32,33]. It is therefore believed that in our case the highly conductive PPy/MWCNT composites can be achieved because the enhanced interaction between PPy and the MWCNTs surface will be favorable to anchoring the PPy backbone onto the MWNTs surface [34].

Normally, there are significant challenges in tuning the thickness of the polymer shell, since it is intractable in processing chemically the synthesized polymer onto the surface of the carbon nanotubes. Fortunately, an ultrathin and strongly adherent polypyrrole shell grown on the surface of carbon nanotubes are readily obtained directly by our improved method. The morphology and the thickness of polypyrrole shells were kept nicely in our reproducible tests, permitting tuning the thickness of polymer shell by changing the mass ratio of Pyrrole monomers to MWCNTs. Therefore, the PPy/MWCNT composites with tunable thickness of polymer shell were easily fabricated.

**Figure 5:**
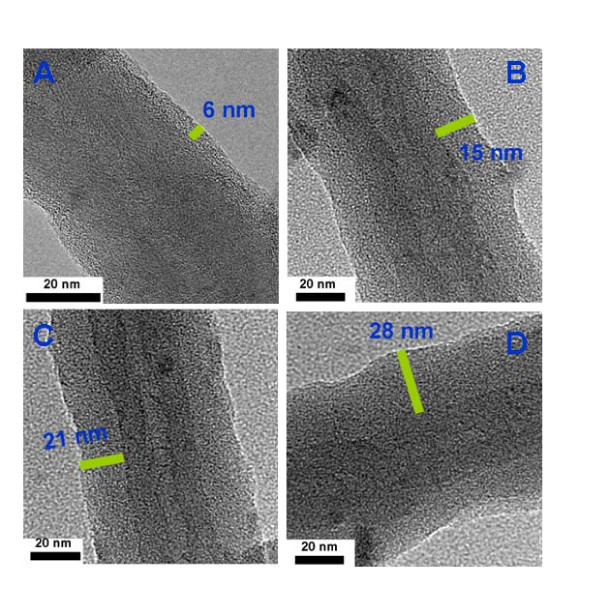
**HRTEM images of PPy/MWCNT composites with different mass ratios of PPy/MWCNT**: (A) 2:8; (B) 4:6; (C) 5:5; (D) 6:4.

Figure 5 presents the TEM images of four PPy/MWCNT samples prepared with various mass ratios of PPy monomer to MWCNT. In Figure. 5A, it is observed that PPy/MWCNT-1, synthesized at a PPy/MWCNT ratio of 2:8, is composed of ultrathin PPy shell coating on the surface of MWCNT core. The MWCNT diameter and PPy thickness are estimated to be around 31.7 nm and 6 nm, respectively. When the ratios of PPy/MWCNT were changed to 4:6 and 5:5, the shell thickness of these two PPy/MWCNT composites are estimated to be around 15.2 nm and 21 nm, respectively (Figures. 5B and 5C). As the ratio of PPy/MWCNT is raised to 6:4, the thickness of PPy shell reaches around 28 nm. Moreover, the surfaces of the four composites are all smooth, uniform and free of any granular product [35,36]. It is interesting to note that the thickness of the polymer is not increased remarkably as reported in the previous literature [21] when the mass ratio of the PPy/MWCNT is changed. The reason may be related to the fact that the polymerization rate of PPy is reduced obviously by adding ethanol in the reaction solution as mentioned above. Importantly, by using the facile synthesis approach, the thickness of PPy shell can be controlled by tuning the mass ratio of PPy monomer to MWCNT accurately, which may provide the favorable choice for the practical synthesis application of conjugated polymer/MWCNT composites.

**Figure 6:**
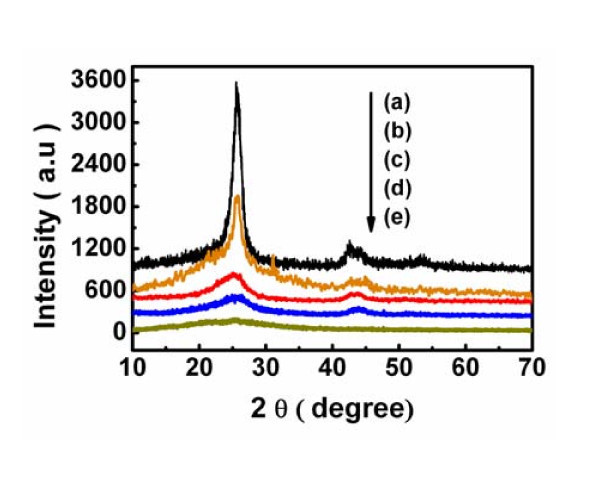
**Comparison of XRD spectra of (a) MWCNTs, PPy/MWCNT composites with various PPy thickness (nm)**: (b) 6; (c) 15; (d) 28, and (e) PPy.

A comparison of the X-ray diffraction [XRD] spectra of different molar mixtures of PPy/MWCNT, MWCNTs and PPy composites are shown in Figure 6. The X-ray pattern of the MWCNT displays the presence of two peaks at 25.80^° ^(3.47 Å) and 42.75^° ^(2.12 Å) assigned to (002) and (100) diffractions corresponding to the interlayer spacing (0.34 nm) of the nanotube and reflection of the carbon atoms, respectively, in good agreement with that of the previous literature [37]. For pure PPy, a broad diffraction peak at 25.4^° ^is due to the pyrrole intermolecular spacing [36]. For the different molar mixtures of PPy/MWCNT, the XRD spectra show both the PPy broad peak (at 25.4^º^) and the strong MWCNTs peaks (at 25.80^° ^and 42.75^º^) [21,22]. It is found that the intensity of MWCNTs diffraction peaks decreases with increasing the mass ratio of pyrrole to MWCNTs but is still stronger than the PPy peaks when the mass ratio of pyrrole to MWCNT reaches 6:4.

**Figure 7:**
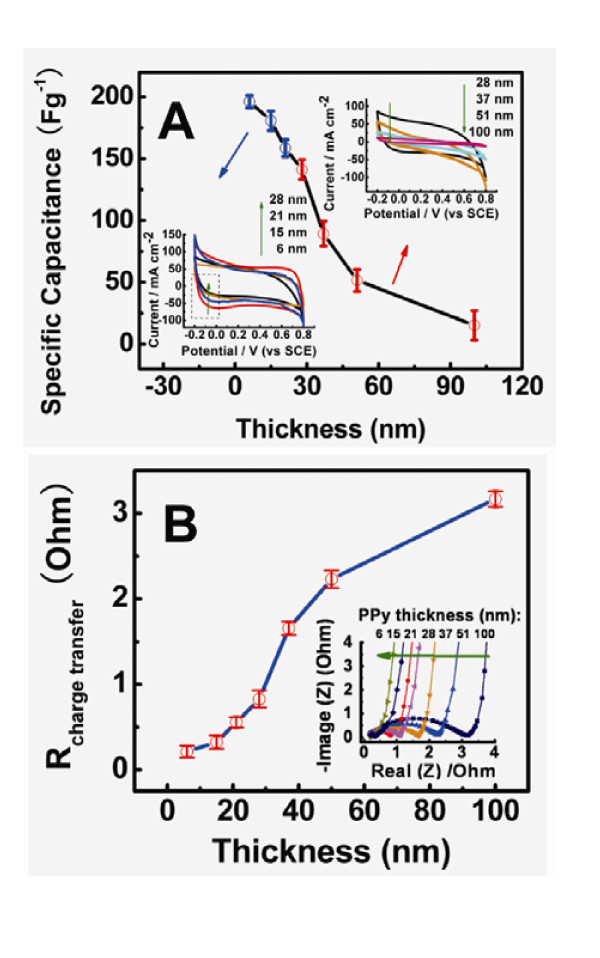
**Capacitance values of CNT/PPy composites**. (A) Specific capacitance of PPy/MWCNT composites with different PPy thickness. The insets are CV curves of PPy/MWCNT composites with various PPy thickness (nm): 6, 15, 21, 28, 37, 51 and 100 nm in 1 *M *KCl solution at scanning rate of 100 mVs^-1^. (B) PPy thickness dependence of the charge transfer resistance of PPy/MWCNT composites. The insets are EIS curves of PPy/MWCNT composites with different PPy thickness.

Furthermore, the electrochemical properties of the PPy/MWCNT composites compared with pristine MWCNTs and pure polypyrrole were evaluated by cyclic voltammetry test. As shown in Figure S3-A in Additional file 1 the electrochemical properties of the PPy/MWCNT composite which the thickness of PPy shell is 6 nm have been obtained by cyclic voltammetry [CV] with different scan rates. They all show the typical double-layer capacity behavior, which benefited from their large surface area [38-41]. It can be found that the CV curves of PPy/MWCNT composite are rectangle-shaped, resulting from a very quick charging/discharging process in PPy/MWCNT composite [32]. Compared with the CV curves of PPy/MWCNT composites, CV curves of both pure PPy and MWCNTs show lower specific capacitance and non-rectangle-shape. Thus it can be confirmed that the electrochemical properties of PPy/MWCNT composites are superior than those of the individual component PPy or MWCNT. (Figures S3-B and S3-C in Additional file 1) [8,42] This can be attributed to the special structure and morphology of the MWCNT-PPy core-shell composite. The long-term cycle stability of the PPy/MWCNT composite with the thickness of 6 nm was also evaluated by repeating the CV test at a scan rate of 200 mVs^-1 ^for 1000 cycles. (Figure S4 in Additional file 1) The PPy/MWCNT electrode exhibits excellent stability over the entire cycle numbers and maintains 73.6% of its initial capacity after 1000 cycles, which is consistent with that reported in the previous literature [38-41]. Swelling and shrinkage of electrochemically active conducting polymers is well known and may lead to degradation of the electrode during cycling. This has been overcome by the core-shell structures, which maybe benefit from the strong interaction between CNT and PPy[38-41]. After several 1000 cycles, the interaction force between CNT and PPy remains unchanged and the PPy shell appears to have a dense sheet structure, which implies that the transfer ability of charges remains fairly constant. Hence, it could be considered that an interesting synergistic effect between MWCNT and PPy plays an important role in the electrochemical charge-discharge process. Firstly, the core-shell structure leads to an increase in the surface area of the PPy/MWCNT composite, which enhances MWCNTs solubility and dispersibility and improves effectively the contact with the electrode and electrolyte. Secondly, the conductivity of the MWNTs dispersed throughout the structure increases the electrical conductivity of the composite film over the entire PPy redox cycle. Thirdly, the ultrathin PPy shell could effectively shorten the transport path of ion diffusion through the solid phase and decrease the contact resistance between the polymer and CNT, which can significantly improve the charge transfer ability between the polymer shell and CNT[8,39]. Therefore, the relationship between the thickness of PPy and the electrical properties of the PPy/MWCNT composites should be taken into account. The specific capacitance values of CNT/PPy composites with different PPy thickness from 6 nm to 100 nm (including 6, 15, 21, 28, 37, 51, and 100 nm) are presented in Figure. 7A. [*Note: In order to collect solid data, for every single sample, we fabricate three electrodes to do the measurements and the average values with error bar are presented in both Figures *7A*and *6B*accordingly*] Clearly, the specific capacitance decreases linearly as the thickness of polymer shell increases. However, when the PPy thickness reaches 28 nm, the nonlinear decrease of the specific capacitance with the increase of PPy thickness is clear. As shown in Figure 7A, the specific capacitance of the PPy/CNT composites decreases with the increase of PPy thickness and the trend is intensified when the PPy thichness is thicker than about 30 nm. Generally, for an ideal electrode material, the response current rapidly reaches a steady-state value due to its high electrical conductivity when the sweep direction of potential is changed, leading to rectangular-shaped CV curves. Hence, the current/potential slope at the switching potentials can be used to qualitatively reflect a magnitude of the active electrode material's conductivity; the steeper the slope, the higher the conductivity[43]. From Figure 7A, the CV curves are not rectangle-shaped gradually at a sweep rate of 100 mVs^-1 ^as the increase of PPy thickness, indicating the resistance-like electrochemical behavior[35]. In the conducting polymer composites, the conductivity depends not only on the doping level or conjugated length but also on some external factors such as the compactness of the sample or orientation of the microparticles [42,43]. Based on this analysis, the change of the electrical performance of the PPy/CNT composite may relate to the synergistic effect of these factors aforesaid. Nevertheless, the beneficial effect will reduce with the increase of the ratio of PPy:CNT. This may be because the thick PPy shell is too compact to hinder counterions entering into/ejecting from the PPy films to reach the surface of CNT.

On the other hand, the specific capacitance of electrochemical supercapacitor depends strongly on not only the rates of ionic mass transport but also the series resistance (R) [34,35,44]. For further understanding the relationship between the thickness of polymer shell and electrochemical properties, the resistance of the PPy/CNT composites are investigated by the electrochemical impedance spectroscopy [EIS], which is another powerful tool for mechanistic analysis of interfacial processes and for evaluation of double-layer capacitance, rate constants, etc [45]. The EIS can be observed as a single and distorted semicircle in the high-frequency region and a near-vertical line in the low-frequency region for both the Nyquist plots. The semicircle portion corresponds to the electron transfer-limited process, whereas the linear part is characteristic of the lower frequencies range and represents the diffusion-limited electron-transfer process [46,47]. It can show all of resistances of supercapacitors, which are the electrolyte resistance (R_s_) and the sum of the electrode itself and the contact resistance between the electrode and the current collector (R_f_). The electrolyte resistance and the contact resistance are identical under the same test condition. Therefore, an increase of R_f _indicates an increase of the PPy/CNT electrode resistance which is represented by the diameter of the semicircle on the Z' axis in impedance plots (Z*plots) [43] Based upon this, as shown in Figure 7B, it is clear that the diameters of semicircle of PPy/MWCNTs composites increase with the increase of PPy thickness, indicating a clear dependence of charge-transfer resistances on the polymer thickness. Therefore, the relationship between PPy thickness and electrical properties of the PPy/CNT composites should include: 1) Thin PPy shell is facile to enter into/eject cations and anions. As the PPy thickness increases, the ionic mass transport becomes slow to reach all the available interfaces between PPy and CNT due to the more compact polymer [34,35]. 2) Compared with Figures 7A and 7B, for PPy/CNT composites with a thinner PPy shell (< around 30 nm), the diffusion-limited electron-transfer process may dominate the electrical properties of the composite because the electrode itself resistance plays a major role in the specific capacitance. Like in a metallic system, the diffusion of the charge carriers is determined by the band structure around the Fermi energy and hence, much information about the electronic band structure of polymer/CNT composite can be obtained [45], However, when the thickness of polymer is thicker than 40 nm, other factors such as the rates of ionic mass transport and compactness of the sample may become more important. Thus, the electron transfer-limited process dominates the electrical properties of the composites because the electron transfer resistance of the polymer/CNTs composites increases with the polymer thickness [45]. Thus, controlling the thickness of the polymer coating on the CNTs plays an important role in functionalizing the CNTs. Furthermore, this approach could provide a more efficient way for further researches in the carbon nanotube based composites.

## Conclusions

In summary, an ultrathin and uniform polypyrrole (PPy) film has been successfully coated on MWCNTs through an improved *in situ *chemical oxidation polymerization. The thickness of the polymer can be precisely controlled by adding ethanol in the reaction system and adjusting the mass ratio of PPy/MWCNT. The possible mechanism is that ethanol has a pivotal effect on controlling the degree of self-polymerization of pyrrole monomers and the morphology of polymer film on MWCNTs surface by restraining the polymerization reaction rate. The thickness of PPy film has a great effect on the electrical properties of polymer/CNT composites. The facile synthesis method may provide a very promising candidate avenue in controlling the morphology of polymers coating on carbon nanotubes, especially in fabricating the desirable performance of electronic devices.

## Methods

### Synthesis process

The milled MWCNTs were carefully separated through 200 mesh screen and then functionalized in 2.6 *M *nitric acid (HNO_3_) at 80°C for 14 h to get abundant carboxyl groups at the defect sites and the end of the nanotubes [48]. Subsequently, the carboxylic acid-functionalized MWCNTs were thoroughly washed with distilled water and centrifuged several times until the aqueous solution reached a neutral pH and left to dry in air. Whereafter, they were dispersed in the mixed solution with 1 *M *HClO_4 _solution and ethanol (V_ethanol_/V_acid solution _= 1:5) followed by 10 min of ultrasonication. Pyrrole monomers were added to the solution and the mixture was vigorously stirred for 30 minutes. The equal molar of ammonium persulfate (APS) dissolved in acid solution was slowly added to initiate the polymerization at 0~5°C. This mixture was stirred by magnetic stirring for 8 h. At the end of the reaction, a litte acetone was added to terminate the reaction. Following the typical preparation, the PPy/MWCNT composites can be prepared with various thickness of PPy shell by changing the mass ratio of pyrrole monomer/MWCNT.

### Characterization

High-resolution microscopy measurements were performed using a JEM-2010HR transmission electron microscope (TEM) with operating voltage of 120 kV. Raman spectra were recorded at room temperature utilizing back scattering mode on a Renishaw inVia system. The 514.5 nm line of an Ar^+ ^laser was used as the excitation resource. A thermogravimetric analysis [TGA] was carried out in a NetzschTG-209 system. The samples were scanned from 0 to 900°C at a heating rate of 10°C/min in the presence of nitrogen. Morphology and microstructure of the as-obtained composites were performed using X-ray diffraction (XRD, Rigaku D/MAX 2200 VPC, Rigaku company, Japan). The cyclic voltammetry was conducted by an electrochemical station (CH Instruments 660 C, Shanghai Chenhua, China) using conventional three-electrode conFigureuration with a platinum sheet as the counter electrode and a saturated calomel electrode (SCE) as the reference electrode [25]. The electrolyte containing 1 *M *KCl dissolved in aqueous solution was deoxygenated under a flow of N_2 _for 30 min. The specific capacitance obtained from the CV curve could be calculated according to the equation C = I/sm, where 'I' was the average current, 's' was the potential sweep rate, and 'm' was the mass of each electrode. The composite electrodes were prepared by dispersing the PPy/MWCNT composites or carbon nanotubes, pure PPy samples and PTFE (5%), followed by adding a small amount of ethanol and NMP to yield a homogenous paste. The paste was spread onto the nickel foam collectors (1 × 1 cm^2^) and then pressed under 10 MPa. These electrodes were dried in vacuum at 60°C for 24 h. Electrochemical impedance spectroscopy (EIS) measurements (excitation signal: 5 mV; frequency range: 100 kHz down to 10 mHz) were carried out using an IME6X electrochemical workstation.

## Competing interests

The authors declare that they have no competing interests.

## Authors' contributions

YX, YZ, LD carried out the synthesis of PPy/CNT composites. BZ, MZ, JY, YC, XC and JZ carried out the characterization of PPy/CNT composites and drafted the manuscript.

## Supplementary Material

Additional file 1 **Electronic Supplementary Material**. Word DOC containing Supplemental Figures S1, S2, S3 and S4 Click here for file
