# Supplementary material for: A Facile Synthesis of Polypyrrole/Carbon Nanotube Composites with Ultrathin, Uniform and Thickness-Tunable Polypyrrole Shells
Source: Nanoscale Res Lett. 2011 Jun 17;6(1):431. doi: 10.1186/1556-276X-6-431 (PMC3211849; doi:10.1186/1556-276X-6-431)
Supplement: Additional file 1 — Electronic Supplementary Material. Word DOC containing Supplemental Figures S1, S2, S3 and S4 [file 1556-276X-6-431-S1.DOC]

# Additional files

### Electronic Supplementary Material


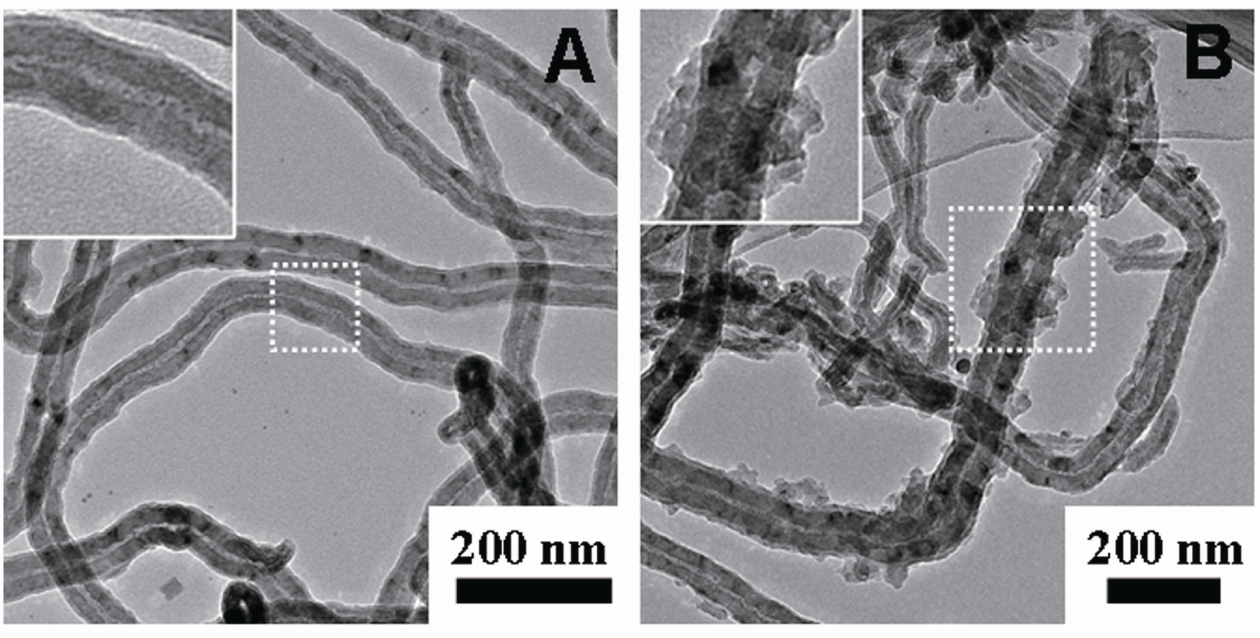


**Figure. S-1.** TEM of the PPy/MWCNT composite synthesized were prepared at the same mass ratio of pyrrole to MWCNTs (2:8) with (A) and without (B) the addition of ethanol in the reaction solution.

Compared to polypyrrole obtained in the mixed solution of V_ethanol_/V_acid solution_=1:5, the PPy/MWCNT composite was synthesized with the mass ratio of Py/MWCNT (2:8) in the mixed solution of V_ethanol_/V_acid solution_=1:1 by the same method. After 8 h of reaction time, there was no PPy film found on the surface of carbon nanotubes. Until the reaction time reached 12 h, the thickness of PPy film reaches 4 nm (**Figure. S2-A**). Over an extended reaction time of 24 h, the polymer thickness increased to 8 nm (**Figure. S2-B**). It is important to note that the PPy films obtained on the CNTs surface are smooth, uniform and free of any irregular nanoparticles or sediments, though the reaction time is prolonged markedly.


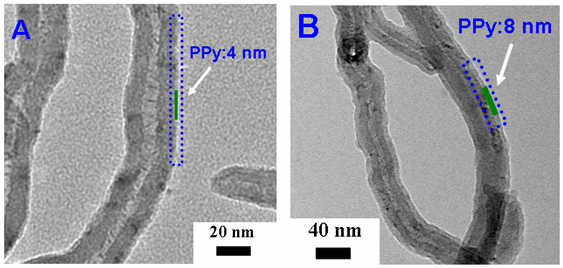


**Figure. S-2.** TEM of the PPy/MWCNT composite synthesized in the mixed solution of V_ethanol_/V_acid_ solution = 1:1 with the mass ratio of Py/MWCNT (2:8). The reaction time: (A) 12 h and (B) 24 h.


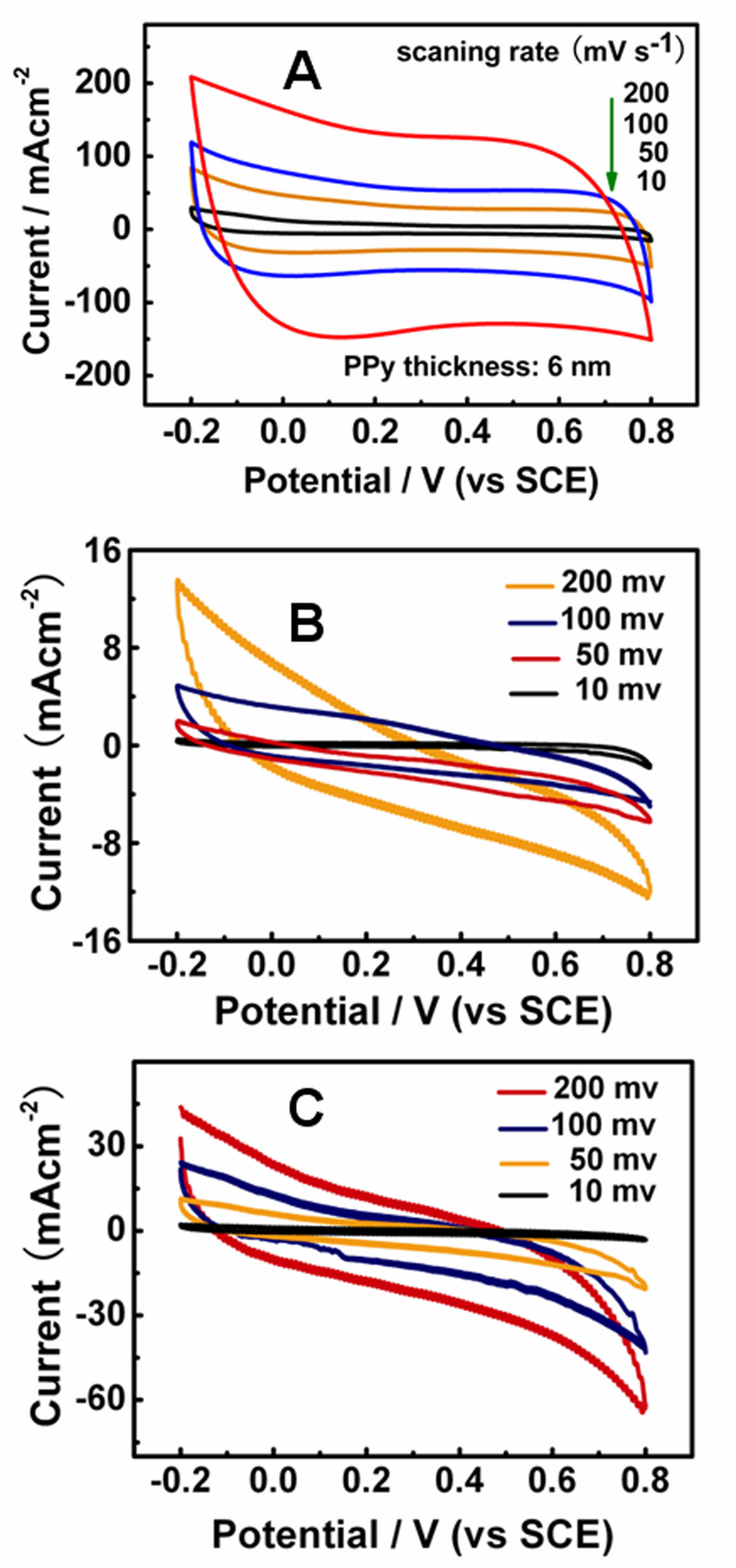


**Figure. S-3.** CV curves of (A) PPy/CNT composite with a PPy thickness of 6 nm, (B) pure PPy films and (C) pristine MWCNTs in 1 *M* KCl solution. at a scanning rate of 10, 50, 100 and 200 mV·s^-1^.


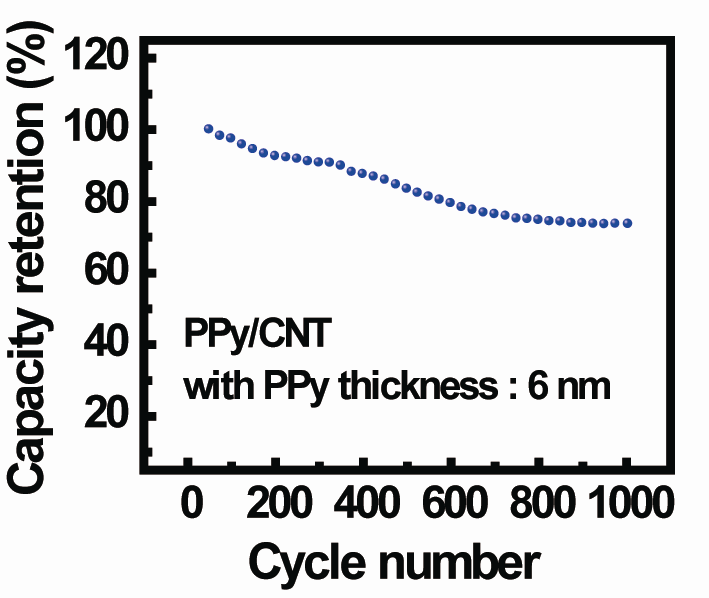


**Figure. S-4.** Variation of the specific capacitance of PPy/MWCNT with PPy thickness of 6 nm as a function of cycle number measured at 200mV·s^−1^ in 1 *M* KCl aqueous solution.
